# Work readiness of pharmacy graduates: An exploratory study.

**DOI:** 10.1016/j.rcsop.2023.100389

**Published:** 2023-12-07

**Authors:** Wei Jin Wong, Ronald F.S. Lee, Li Yun Chong, Shaun Wen Huey Lee, Wee Ming Lau

**Affiliations:** a School of Pharmacy, Monash University, Malaysia; b Jeffrey Cheah School of Medicine and Health Sciences, Monash University, Malaysia

**Keywords:** Work-readiness, Pharmacy education, Covid-19 impact, Health workforce development

## Abstract

**Introduction:**

The recent global pandemic of Covid-19 caused various disruptions. Among them were face-to-face teaching and learning activities being switched to virtual sessions in accordance with health authorities recommendations. The impact of these changes on work readiness of pharmacy graduates is unknown.

**Aim:**

This study aims to determine the impact of pharmacy graduate's work readiness, particularly those that had their studies disrupted from the pandemic.

**Methods:**

Practicing pharmacists with supervisory experience were interviewed on their opinions on work readiness of early career and intern pharmacists. Specifically, they were asked to comment on work readiness of pharmacy graduates who had their later stage of pharmacy education impacted by the pandemic. Data was transcribed verbatim and thematically analysed. This was also supplemented with quantitative data from graduating students in 2020 and 2021 using the Work Readiness Scale.

**Results:**

Qualitative feedback showed four themes related to workforce readiness: work competence, social intelligence, personal characteristics, and organizational acumen. Preceptors interviewed noted differences in communication abilities when interacting with patients. However, this improved with time. Quantitative data collected from graduates via the validated Work-Readiness Scale also showed a more positive agreement towards perceived work readiness. These graduates were comfortable with using technology as they had used these extensively in their learning during the pandemic and thus was comfortable in adopting digital health tools in their practice.

**Conclusion:**

Although graduates reported to be work ready, there were gaps in communication skills and confidence levels when interacting with patients, as reported by supervising preceptors. Graduates also described this sense of ‘missing out’ from not having the opportunity to attend face-to-face activities like their originally planned hospital placements and how it impacted their choice of career. As pharmacists continue to play vital roles as members of the broader healthcare workforce, both in clinical and nonclinical settings, learnings from this study should be considered in designing educational activities to train and develop the workforce of the future.

## 1 Introduction

Pharmacists as healthcare professionals play vital roles as members of the broader health workforce. Globally, health systems are facing shortage of healthcare workers. This is also true for less-resourced low to middle-income countries like Malaysia.^1^ In these settings, the contribution of existing pharmacists then becomes more needed and valued. This was especially apparent while managing the global pandemic of Covid-19.^2^

In Malaysia, there is a dual system of public and private healthcare and about a third of registered pharmacists work in the private sector.^3^ Apart from the public and private hospitals, pharmacists also practice in community pharmacy settings and public community clinics. Currently, there are around 18,000 registered pharmacists in Malaysia.^4^ Estimates found that the overall population to community pharmacy ratio was 1:9000, lower than the suggested ratio for high-income countries of 1:2000–8000.^5^ There is still a need for more pharmacists as Malaysia desires to be a developed nation with world class health systems. This highlights the critical need for continual training and development of competent pharmacists.

In the Malaysian context, pharmacy education occurs within both private and public based institutions and upon graduation, students need to complete a 1-year internship to obtain full registration.^6^ Face-to-face teaching and learning activities such as clinical placements, case-based learning and Objective Structured Clinical Examination (OSCE) activities are components of current undergraduate programs that are used to help train and prepare graduates. These are important as, in addition to using technical knowledge to make decisions, graduates are expected to be able to communicate clearly, show leadership and exercise empathy when interacting with patients. Teaching institutions thus incorporate development and assessment of these soft skills into their curriculum as part of their program.

The need for a myriad of hard and soft skills, as well as the challenging healthcare working environment can cause unnecessary burdens and stress on graduates entering the workforce. This can be an issue particularly if they are not well prepared for these challenges, which could be detrimental to both them and their patients. A high level of work readiness can help encourage a smoother transition and provide the individual with the confidence to navigate through the uncertainties that are linked to transitioning from university to work settings, particularly against the backdrop of an ever-evolving healthcare environment. However, there is a paucity of work done in evaluating this in healthcare graduates. In addition, most studies focus on medical^7^ and nursing graduates^8^^,^^9^ with some work done on mixed groups of healthcare disciplines^10^ but very few on pharmacy graduates.^11^^,^^12^

The recent global Covid-19 pandemic could have influenced levels of work readiness in pharmacy graduates due to changes to the educational system induced by global lockdowns.^13^ For graduates-to-be, their learning experiences were mainly affected with face-to-face sessions being switched to virtual learning. Additionally, the added social and psychological impact from quarantined measures and other related precautions may have influenced their mental health and development.^14^ Students graduating in uncertain times like the global pandemic may also face heightened anxiety due to the unprecedented situation.^15^ Effects of the disruption from the global pandemic is only now beginning to be slowly noticed in different sectors.^16^^,^^17^ There are limited studies that specifically sought to explore impact on how the work readiness of pharmacy graduates were affected by this pandemic.^18^

In this exploratory study, we sought to examine what were the effects of Covid-19 induced educational changes on the work readiness of pharmacy graduates in Malaysia. To achieve this aim, we utilized (1) surveys to examine the perception of recent pharmacy graduates on their work readiness and, (2) interviews with pharmacy preceptors who are involved in supervising pharmacist interns and early career pharmacists.

We foresee that the results of this study will better inform educational institutions and policy makers on the strengths and weaknesses of the educational changes done during the pandemic and its effects on the student populations that were affected. In addition, the study findings could guide efforts to aid the successful integration of these affected student cohorts into the workplace.

## 2 Methods

### 2.1 Work readiness scale

The Work Readiness Scale (WRS) for health professional graduates was utilized as a basis for surveys and interviews in this study. The WRS has been validated in final-year health professional graduates in Malaysia.^19^ It contains 53 questions/statements that looked at various aspects of work-readiness: namely work competence, social intelligence, personal characteristics and organizational acumen. Studies have been taken to review and validate its usefulness in various settings with health professional graduates of different backgrounds.^8^^,^^19^^,^^20^ It also has been used to explore work readiness of pharmacy graduates in other settings.^12^

### 2.2 Interviews (preceptors)

Pharmacist preceptors were chosen as they would be constant in providing supervision for the different cohorts of graduates. Study participants were recruited from relevant healthcare employment institutions that represent the bulk of employment for pharmacy graduates in Malaysia. These institutions include private and public hospitals, government health clinics, and community pharmacies. Inclusion criteria for this group of study participants: they would be currently registered and are practicing pharmacists themselves, who play a supervisory role in mentoring early career pharmacy graduates. Recruitment was done via convenience sampling through personal contact. Semi-structured interviews were conducted online by a research assistant in the presence of one of the study investigators. The four work readiness factors from the WRS^20^ were used to guide the semi-structured interviews. Preceptors were asked to compare their perspectives on the work readiness of recent graduates who had undergone curricula changes in their final year due to COVID-19 as compared to their counterparts who graduated earlier. All interviews were recorded and transcribed verbatim. Transcriptions were cross-validated by one of the study investigators. All participants who took part in the interview were awarded a RM50 gift voucher.

### 2.3 Survey of pharmacy graduates

Pharmacy graduates who had their final year of studies impacted by the disruption from the Covid-19 pandemic and graduated in the year of 2020 or 2021 were also invited to take part in an electronic survey based on the WRS. A 4-point Likert scale, with scores of 1 (strongly disagree) to 4 (strongly agree) was used, as per the validated version. An open-ended question was appended at the end of the survey (to a limit of 200 words), *‘Please comment on how your work readiness would have differed, if there was NO Covid-19 pandemic and learning activities/clinical attachments proceeded as per normal.’*

The survey was created and administered using Qualtrics. It was advertised through professional societies, early career groups and student alumni societies. In addition, snowball sampling was performed via contact with alumni, and pharmacy preceptors. The survey was administered from June 2020 to August 2021. Participants who completed the survey went into a random ballot for a RM50 gift voucher.

This project received ethics approval, Monash University Human Research Ethics Committee (MUHREC), project ID = 28,139.

### 2.4 Data analysis

#### 2.4.1 Qualitative data

Qualitative data gathered from surveys and interviews were coded against the WRS framework as themes. First, familiarization with the transcribed data was done by listening to audio recordings and reading transcripts. The transcripts were then coded by three study investigators with the aid of NVivo, version 1.7 (QSR International). The codes were then reviewed together to resolve any discrepancies and generate consensus on the comparison between the pre-pandemic and pandemic batches for each theme and sub-theme. This was done iteratively with each interview, and we continued interviews until we did not obtain any new information. Data saturation was achieved after 9 interviews. Themes were presented with illustrative quotes affixed with participant code (graduates) or healthcare setting (preceptors).

#### 2.4.2 Quantitative data

Fully completed surveys were included for analysis. Descriptive statistics were applied for sociodemographic background of both survey and interview participants, and also for survey responses, using IBM SPSS Statistics version 26 (IBM Corp., Armonk, N.Y., USA). In the WRS survey, the scoring was 1 = strongly disagree, 2 = disagree, 3 = agree, 4 = strongly agree. However, some statements were worded such that high agreement signified lower work readiness. Thus, we reversed the scores for these statements and the mean scores were calculated across all 53 statements where a score of 4 represents high work readiness and a score of 1 represents low work readiness. (Refer Appendix D).

## 3 Results

### 3.1 Qualitative analysis (feedback from preceptors)

A total of 9 interviews were conducted with pharmacy preceptors until we reached data saturation based on the pre-determined themes. Demographics of the preceptors are summarized in Table 1. Most of the preceptors were female, between the age of 30–40 years. Most of them have >10 years of experience working as a pharmacist (*n* = 5; 55.6%), and >5 years of experience working as a preceptor (n = 5, 55.6%). The preceptors were from different healthcare setting; 4 (44.5%) from community pharmacy (CP), 3 (33.3%) from government hospital (GH), 1 (11.1%) from private hospital (PH), and 1 (11.1%) from government health clinic (GHC).

**Table 1:** Demographic Data of Pharmacy Preceptors.

	Number	Frequency (%)
Age (Years)		
30–40	8	88.9
40–50	1	11.1
Gender		
Male	2	22.2
Female	7	77.8
Years of experience as a pharmacist		
≤5	0	0
6–10	4	44.4
11–15	4	44.4
≥16	1	11.2
Years of experience as a preceptor		
<5	4	44.4
6–10	5	55.6
Healthcare setting		
Community pharmacy	4	44.5
Government hospital	3	33.3
Private hospital	1	11.1
Government health clinic	1	11.1

The four major domains of work readiness were explored in the interview with preceptors, as shown in Table 2 (work competence, social intelligence, personal characteristics and organizational acumen).

**Table 2:** Factors of work readiness for healthcare graduates.

**Work Competence**	**Social Intelligence**	**Personal Characteristics**	**Organizational Acumen**
Clinical knowledge Clinical skills⁎ Confidence⁎ Responsibilities	Communication⁎ Teamwork Managing interpersonal conflict⁎	Resilience Flexibility Stress management	Ward knowledge Knowledge on workplace policy and procedures Personal development opportunities

⁎ Components where there were noticeable differences between the graduates.

**Fig. 1:**
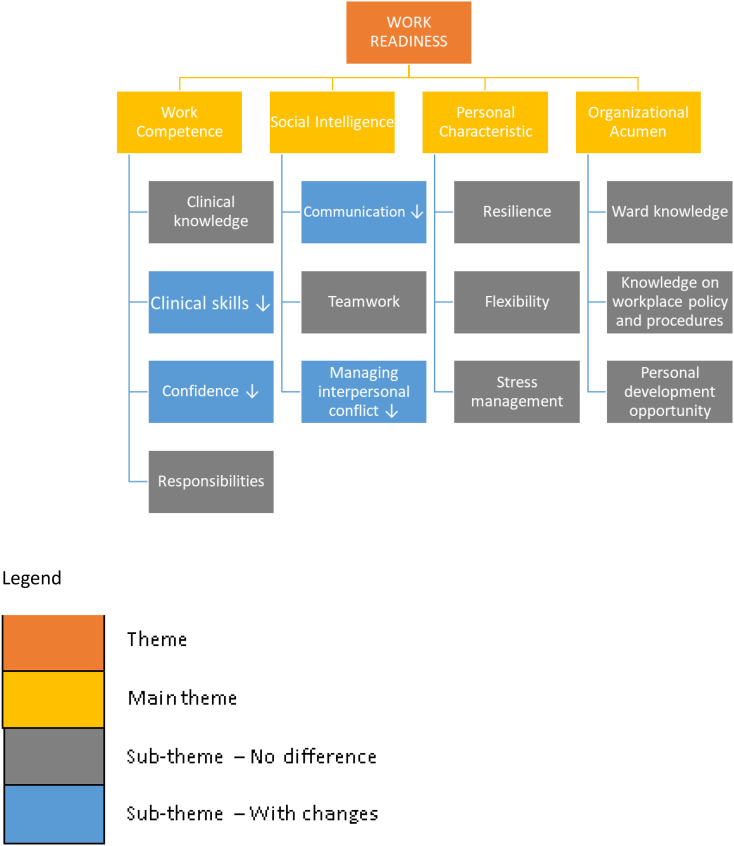
Flowchart of the four factors of work readiness.

Semi-structured interviews were conducted with pharmacy preceptors using the four domains of the WRS scale as a guide. Their opinions were sought on recent pharmacy graduates (affected by the pandemic during undergraduate learning) compared to previous batches (unaffected by pandemic). Fig. 1 summarizes this and the differences between the graduates. Appendix A lists the different themes and quotes shared by preceptors.

### 3.2 Work competence

Overall, the preceptors found no difference in the clinical knowledge of the recent graduates compared to before.

However, the clinical skills of recent pharmacy graduates had been affected. Due to the lack of clinical exposure, they find it harder to apply their theoretical knowledge to clinical practice, and therefore impacted their clinical reasoning. For example, they may fail to relate patient's clinical presentation to a particular medical problem, even though they know the signs and symptoms of the medical problem. In addition, they sometimes take drug information directly from references without correlating this to other clinical parameters of patients to make better clinical decisions.

*If it's renal adjustment dose, sometimes they take information directly from the package insert or from the references, they don't look at the patient clinically. For example, if the creatinine clearance is between 30 and 50, then there's a specific frequency that they have to adjust to, but they don't see the patient's urine output. You don't [always] have to adjust following the reference. You can suggest a higher frequency or a higher dose because patient still has urine. I think they need a bit of experience going to the ward and seeing more cases. (P6, GH).*

The preceptors also found that the recent graduates had lower confidence in various aspects which differed based on their practice setting. From the community pharmacy practice angle, the weaknesses were in aspects of health screening and counselling, in handling prescription related issues, handling patient complains and in functioning independently. In government hospitals this lack of confidence manifested especially in communication with doctors where they were hesitant to approach for medication related discussions.

*For this batch, they will be stuck and don't know what they need to do on the spot. Although we know that we need to call the doctor immediately, they are still asking their preceptor, or colleague, to call the hospital and confirm with the doctor. Actually, they are the one who needs to call to confirm with the doctor, but they don't dare or are shy, They also don't like to talk to other healthcare professionals. (P4, CP).*

There was no difference in terms of responsibility where Pharmacy supervisors agreed that responsible attitudes are more a product of upbringing than university education.

### 3.3 Social intelligence

For the batch of recent Pharmacy graduates, communication skill had been affected where they were less comfortable talking to colleagues and patients or customers, had less active listening and less effective communication skills when interacting with patients during clinical assessment and counselling. These could be attributed to the shortened internship durations during their studies due to the pandemic.

*Because of the lack of experience, for example, they have to shorten their internship, or during the housemanship or internship or attachment in a community pharmacy, they won't be able to talk to a customer as easily as before. (P1, CP).*

Clinical skills that involve specialized communication skills, such as history taking and counselling, were also affected. For example, they were less able to probe the patient for relevant information or summarize the important information during history taking. As exemplified by one pharmacist:

*They like to talk, but they are less proactive in capturing what was inside the conversation, important points. They cannot summarize what was communicated to them. But the previous batch they can catch up very fast, oh this customer, or this patient got hypertension, diabetes. Sometimes it's through chit chat with the customers. But we need to summarize the chit chat into the medication history. The previous batch do this better. (P4, CP).*

In terms of counselling, some recent graduates had a disorganized or messy counselling approache which necessitated additional supervision compared to previous batches. In addition, there was an increased use of medical jargon instead of layman's terms when explaining a condition or a drug to the patients. This was particularly an issue in rural settings where patient education levels were lower as exemplified by one hospital Pharmacist:

*…because they didn't really meet the patients much as compared to the previous batch, so the way that they speak is really from the book, it's not “live” enough. [......] Especially in our Sabah [a state in East Malaysia] population, our education level is not so good, so we have to speak in a very layman's term. We cannot speak too in-depth using jargons. (P7, GH).*

In community pharmacy, it had been noted that recent pharmacy graduates had a lower ability to manage interpersonal conflict. One potential cause was the change of environment in the community setting post pandemic, where there were more frustrated customers, and so the requirement for them to manage customer conflict has increased. Another potential reason is the lack of confidence to manage customer conflict which resulted in them passing over the responsibility to senior colleagues or their preceptor.

*For frustrated customers, they will pass to the preceptor......The previous batch is slightly better. They will explain to customer, “Uncle, we ask you this question because we care for you, we don't want you to suffer any side effect of the medication”. But this batch, they will straightaway come to the preceptor. (P4, CP).*

Overall, the ability to work in teams was minimally affected, where the new pharmacists were respectful to pharmacy technicians and able to work well with colleagues.

### 3.4 Personal characteristics

Overall, preceptors identified that there were minimal education induced differences in terms of key personal characteristics such as resilience, flexibility and stress management that were related to the pandemic. Generally, most of these elements were attributed more to individual personality traits than anything induced by pedagogical changes from the pandemic. In fact some commented that considering the pandemic induced changes to practice, that the current batch of trainees have shown good resilience.

*Resilience wise, given the situation, I think they are good, because with even the changes [challenges of the COVID-19 pandemic] they are willing to face it, they are able to do a good job in general. So yeah, their resilience is good. (P9, CP).*

### 3.5 Organizational acumen

In general, we found that for pharmacy graduates during the pandemic, ward knowledge and knowledge on workplace policy and procedures had not been affected. This could be attributed to this being very specific where policies and procedures differ from workplace to workplace, and that organizational acumen is something that improves over time the longer one stays within an organization.

*Workplace policy in terms of if you're comparing batches to batches, it is about the same because every workplace will have a different policy. (P1, CP).*

### 3.6 Lack of career clarity

Some of the preceptors we interviewed echoed similar sentiments where recent graduates were unsure of their career choice due to the lack of attachment in hospital, community or industrial pharmacy. This resulted in some of them choosing the wrong career and giving up halfway.

*For the pandemic batch, I am concerned because they didn't have attachment in hospital or community. During interview, they do not know whether they prefer to be in the community pharmacy, hospital or industrial….. I found that graduates don't know their direction well and they didn't have any experience in terms of what to choose for their pathway or future. (P2, CP).*

### 3.7 Quantitative analysis

A total of 30 pharmacy graduates completed the survey (see Appendix C). The participants were mainly female Chinese, with a mean age of 23.73 + 0.980 years. Among these graduates, 21 (70%) graduated in 2020 and 9 (30%) graduated in 2021. Majority of study participants (*n* = 20; 66.7%) have started working as provisionally registered or intern pharmacists at the time of survey completion. In Malaysia, pharmacy graduates start working as provisionally registered pharmacists prior to obtaining full registration as regulated by the Pharmacy Board of Malaysia. Out of the 30 respondents, 21 (70%) respondents provided further feedback on their work readiness through the open-ended question (see Appendix B).

The overall mean score of the 53 statements was 2.93 which is above the midpoint score of 2.50 suggesting that graduates have a slightly positive perception towards their overall work readiness after having their studies affected by the pandemic. Considering the domains of work readiness i.e. work competence (clinical knowledge and confidence) and social intelligence (communication and managing interpersonal conflict) were considered graduates' weak areas by interviews with preceptors, we sought to compare these domains in the graduates' surveys.

We find that on their work competence in terms of clinical skills and confidence in handling practice scenarios, graduates' perceptions on items covering these were slightly positive.

*“I am confident about my learnt knowledge and could readily answer technical questions about my field” (mean score of 2.50).*

*“I have a solid theoretical understanding of my field of work”* (2.67).

*“I feel confident that I will be able to apply my learnt knowledge to the workplace”* (2.90).

*“Now that I have completed my studies I consider myself technically competent to apply myself to the field” (2.70).*

In terms of managing interpersonal conflicts, generally, graduates had a neutral perception of their abilities in this area.

“I feel confident to address interpersonal conflicts at work” (2.50).

“I feel confident to ask for support in dealing with interpersonal conflicts at work” (2.67).

Graduates seem to have a slightly positive perception of their communication abilities.

*“One of my strengths is developing relationships with people”* (3.03).

*“I communicate effectively with different patients or clients”* (2.83).

*“Others would say I have an open and friendly approach”* (3.03).

*“Adapting to different social situations is one of my strengths”* (3.00).

Graduates feedback.

The pharmacy graduates who had their final year impacted by the Covid-19 did not have the opportunity to undergo face-to-face clinical attachment in the hospital. From the responses shared, graduates described this sense of missing out. Many felt that they missed out on clinical experience, exposure, and learning opportunities that the face-to-face clinical attachment provides. They felt that they would have better work readiness had they been able to do clinical attachment, in terms of clinical application and confidence, in addition to being able to communicate more effectively. For example, one student commented that:

*I think I would be more prepared and confident as I step out to work if our placements are arranged as per usual without the pandemic. Honestly placements really help to gain better insights and allow us to immerse in real life settings. Absence of placement makes it quite hard to apply theory to practice as we don't see real life cases. (S21).*

Some respondents also noted that the lack of clinical attachment had influenced their career choice, whereby they will shun away from hospital pharmacy.

*Because of the lack of clinical attachments, I'm swayed away from working in hospital. (S20).*

## 4 Discussion

There is a lack of studies on work readiness of pharmacy graduates especially in developing countries such as Malaysia. Work readiness studies are useful in aligning educational institutions with industry needs and in the context of this study, to inform on the critical issues faced by graduates due to the Covid-19 pandemic and suggests potential steps to aid the integration of future graduates into the workforce.

In domains of the WRS examined during interviews with preceptors, there were no differences found in personal characteristics and organizational acumen in the graduating pharmacy cohort whose studies were affected by the Covid-19 pandemic. However, preceptors noted a reduction in work competence in terms of clinical skills and confidence with a similar reduction in social intelligence in terms of communication and conflict management. In contrast, similar evaluation of final year business students' found them being agile in adapting to using technology to aid communication.^21^ In the study, students also reported acquiring new skills such as technological-related tasks like using video technology and industry specific software programs. However, only students perspectives were examined and its findings should be interpreted in that context.

Graduates also commented on how the lack of face-to-face experiences at placement sites made them feel nervous about their clinical skills and felt a lack of confidence affecting their communication. Similarly, clinical skills were also identified as another area of lack by pharmacy graduates in New Zealand during their internship.^22^ Communication skills is an important component of work readiness and in pharmacy education, various steps have been taken for this to be developed.^23^ Communication skills are also part of the competencies and learning outcomes in pharmacy education.^24^ Face-to-face teaching and learning activities are designed to help pharmacy graduates to develop communication skills. The lack of these in the graduating class affected by the pandemic meant they did not have the same opportunities to develop their communication skills as an undergraduate student. Although so, they were able to improve on it while ‘on the job’ as noted by the interviewed preceptors that their communication abilities did improve with time.

The impact of having virtual sessions, although considered equivalent, may have had some impact on graduates' abilities when communicating with customers/patients. This was highlighted by preceptors which acknowledged the challenges faced by graduates in managing conflicts. In developing patient care skills, the ability to work collaboratively, particularly in multi-disciplinary settings is vital in improving patient care.^25^ The face-to-face interactions with preceptors as a final year healthcare professional student can help graduates' communication skills and in turn improve work readiness.^26^ Going forward, any future curriculum design should consider these factors in preparing health workforce of tomorrow. A feedback loop to workforce development needs and how teaching institutions can help fill the gap should continue to be explored.^27^

For the quantitative surveys on graduates, since we had a limited sample size of 30 participants, we chose to do descriptive reporting of the results to contrast preceptors' views on graduates' work readiness with graduates own personal perceptions. This was sufficient considering the exploratory nature of the study, though a follow up larger scale study would be prudent. Interestingly, we found that scores from the surveys on graduates did not necessarily align to that of preceptor's views, where students often felt neutral to slightly confident in domains that preceptors find them weak in. Though a small difference, the tendency for those relatively new to a domain of skill to overestimate their abilities has been reported previously.^28^ However, as stated above, in the qualitative survey feedback, some students had similar concerns as preceptors on clinical skills, confidence and communication due to lack of face-to-face clinical attachments.

An interesting observation from this study was graduates' perception of missing out on originally planned face-to-face activities like hospital placements and its impact on career choices. Graduates did go through virtual equivalent sessions during their undergraduate education, which were prerequisites of the pharmacy programs and lockdowns meant virtual sessions were the most viable alternatives. Virtual simulations have a place in pharmacy education but should not be the sole learning modality that a student goes through, but complemented with face-to-face activities.^29^ One of the interviewed participants, a practicing community pharmacist also reflected how graduates made decisions in choice of work settings based on experiences during their undergraduate days. This highlights the significant role of experiential learnings at placement sites in shaping initial career choices in graduates.

### 4.1 Strengths

A major strength of the study is that work readiness was explored from both the angles of the graduates themselves and supervising preceptors. The majority of published studies reported on students' or graduates' self-perceived assessment. Since graduates themselves have little experience of pharmacy practice, their views may not reflect the needs of the workplace environment, thus a more balanced view from experienced pharmacy preceptors who actively supervise students provided a more holistic view of graduates' work readiness.

### 4.2 Limitation

Some limitations of the study include the relatively small sample size, comprising 9 interview participants and 30 surveys, the use of a purposive sample for interviews, and the potential for selection bias as graduates who participated may have been more sensitive or aware of the situation. These could have affected the generalizability of the results due to the nature of the participants sampled and that they may not adequately represented the whole pharmacy student population limiting the precision of findings. However, as an exploratory study, the findings highlighted some potential issues with work readiness of pharmacy graduates during the pandemic which warrants carrying out a larger study. It would also be useful to conduct follow up studies on how graduates adapted to the work environment and what they thought was useful in helping them to adjust.

## 5 Conclusion

This study explored work readiness of pharmacy graduates amidst educational changes caused by the global pandemic of Covid-19. Overall, both graduates and supervising preceptors concurred that pandemic-induced changes did not significantly impact the overall work readiness of pharmacy graduates. Nonetheless, some differences in communication abilities, clinical skills and managing interpersonal conflict were observed. This was likely linked to reduced face-to-face experiential placements that did not happen due to the pandemic. Additionally, the lack of placements also possibly influenced graduates' career choice certainty and preferences. Work readiness studies are useful in helping to align educational programs with industry needs. Input from supervising preceptors provides useful feedback as they would be most up to date on current practice needs and able to reflect on interactions with the different batches. Going forward post-Covid-19 pandemic, future work readiness studies can further explore how preceptors can contribute to improving work readiness of graduates.

## Funding

This project was funded by Monash University Malaysia Learning & Teaching Grant 2021 by Education Excellence – STG – 000043.

## Declaration

An abstract was presented at the Asia Pacific Medical Education Conference 2023, National University of Singapore, May 2023.

## CRediT authorship contribution statement

**Wei Jin Wong:** Conceptualization, Data curation, Formal analysis, Funding acquisition, Project administration, Writing – original draft, Writing – review & editing. **Shaun W.H. Lee:** Conceptualization, Formal analysis, Funding acquisition, Project administration, Supervision, Writing – review & editing. **Wee Ming Lau:** Conceptualization, Formal analysis, Funding acquisition, Project administration, Supervision, Writing – review & editing. **Li Yun Chong:** Data curation, Formal analysis, Investigation, Project administration, Writing – original draft. **Ronald F.S. Lee:** Conceptualization, Data curation, Formal analysis, Funding acquisition, Project administration, Writing – original draft, Writing – review & editing.

## Declaration of Competing Interest

The authors declare that they have no known competing financial interests or personal relationships that could have appeared to influence the work reported in this paper.

## Figures and Tables

**Table:** 

Theme	**Sub-theme**	**Sample quotes**
Work competence	Clinical knowledge	*In terms of this pandemic, I don't think that there is any difference in terms of knowledge level. (P5, GH)* *Their clinical knowledge is good and some of them excel well. (P3, PHP)* *[Their baseline clinical knowledge] is almost the same. (P8, GHC)*
	Clinical skills	*What I see is that they need more time in terms of guiding them how to apply their knowledge that they learn in lecture classes into clinical practice. (P5, GH)* *If it's renal adjustment dose, sometimes they take information directly from the package insert or from the references, they don't look at the patient clinically. For example, if the creatinine clearance is between 30 and 50, then there's a specific frequency that they have to adjust to, but they don't see the patient's urine output. You don't [always] have to adjust following the reference. You can suggest a higher frequency or a higher dose because patient still has urine. I think they need a bit of experience going to the ward and seeing more cases. (P6, GH)* *I gave them a set of questions to do, [......] when I discuss with them [recent graduates], they only focus on the question itself. But other PRPs [provisionally registered pharmacists] right, they are, maybe because they are less in the online session, and they have more practical session, that's why they know what I expect them to do. But this batch, I feel like they are all too theory oriented, when you ask them A, they only answer A. Because in clinical pharmacy, you cannot only talk about A, you have to expand and know the patient's data, demographics, and also to find out more details about the patient and drug characteristics. Not just focus on one particular issue. (P7, GH)*
	Confidence	*When doing their attachment in the community pharmacy or in the hospital, because their preceptors want to protect them from being in contact [to COVID-19], there is less chance for them to communicate people, so this gives them lower confidence in terms of health screening, and counselling. (P1, CP)* *For this batch, they will be stuck and don't know what they need to do on the spot. Although we know that we need to call the doctor immediately, they are still asking their preceptor, or colleague, to call the hospital and confirm with the doctor. Actually, they are the one who needs to call to confirm with the doctor, but they don't dare or are shy, They also don't like to talk to other healthcare professionals. (P4, CP)* *Even for simple questions, they [recent graduates] are a bit scared to approach the doctor. For previous batches [of graduates], if I asked to please help me to get countersign of the specialist for some specific medication, they will be very relaxed and help obtain the signature. But this batch they would be reluctant and find it difficult to open their mouth. So I need to help them a bit and push them a bit. (P5, GH)* *The way they speak to doctors makes me think they have a lack of confidence. (P7, GH)* *They will pass to us, the frustrated customer, they will pass to the preceptor…..The previous batch I would say is slightly better…They explain to customer, “Uncle, we ask you this question because we care for you, we don't want you to suffer any side effect of the medication”…..But this batch, they will straightaway come to the preceptor. (P4, CP)*
	Responsibility	*For the responsibility part, I think, its mostly an individual thing. It is not so much correlated with education change or education impact by COVID-19. (P4, CP)* *Their responsibility levels are quite similar. To me this is more to personal attitude. So, no difference to me. (P5, GH)*
Social intelligence	Communication	*Because of the lack of experience, for example, they must shorten their internship, or during the housemanship or internship or attachment in a community pharmacy, they won't be able to talk to a customer as easily as before. (P1, CP)* *They are less proactive in listening to problems not only from healthcare professionals and colleagues but including customers. (P4, CP)* *They like to talk, but they are less proactive in capturing what was inside the conversation, important points. They cannot summarize what was communicated to them. But the previous batch they can catch up very fast, oh this customer, or this patient got hypertension, diabetes. Sometimes it's through chit chat with the customers. But we need to summarize the chit chat into the medication history. The previous batch do this better. (P4, CP)* *For both the current and previous batches, they know the dose, the indication, everything, but when it comes to explaining to the patients, some of the PRPs in this batch will use some jargons, or terms that only medical professionals will understand. It's not layman's term, that's why there's some difference there. In a real practice setting you must use layman's term when you communicate with the patient. (P4, CP)* *For one example, let's say frusemide 40* *mg OD, if you tell the customer or the patient, “uncle, you take frusemide one tablet OD”, the customer or patient will say “what it means by OD?”. That's why you have to say once daily, you cannot say BD or TDS to the patient. (P4, CP)* *…because they didn't really meet the patients much as compared to the previous batch, so the way that they speak is really from the book, it's not “live” enough.…. Especially in Sabah [a state in East Malaysia] population, our education level is not so good, so we have to speak in a very layman's term. We cannot speak too in-depth using jargons. (P7, GH)* *They are just explaining their knowledge, but when it comes to discussing or explaining or counselling patients, I mean when their target audience is pharmacist and their target audience is patient, you can see a difference. Their counselling points can sometimes be a bit of messy, and then we need to stay beside them and always supervise what they say to the patient and correct them after counselling. (P5, GH)*
	Teamwork	*They offer good teamwork. Yeah, even not just among their peers, but with their subordinates, like pharmacy technicians. They are also very much respectful of the pharmacy technicians as previous, they are able to blend in well with them. (P3, PHP)*
	Managing Personal Conflict	*For frustrated customers, they will pass to the preceptor......The previous batch is slightly better. They will explain to customer, “Uncle, we ask you this question because we care for you, we don't want you to suffer any side effect of the medication”. But this batch, they will straightaway come to the preceptor… (P4, CP)* *The interpersonal conflict management is actually similar, but now the requirement for them to manage an interpersonal conflict has increased…..So, so this is what I mean, that in terms of communication and interpersonal conflict management, okay, I would say that there still is more or less similar as the previous batch, but because the needs and the requirement and our expectation for them has increased, so it may seem that it is a little bit lesser, but that is not their fault actually, it is more on the changes of the environment. (P1, CP)*
Personal characteristics	Resilience	*Resilience wise, given the situation, I think they are good, because with even the changes [challenges of the COVID-19 pandemic] they are willing to face it, they can do a good job in general. So yeah, their resilience is good. (P9, CP)*
	Flexibility	*On flexibility, it depends on which PRPs, some PRPs are quite flexible and they are willing to contribute and willing to learn more and willing to do more. (P2, CP)* *Some of them adapt to changes quite fast. This might be human nature that on some learning side, they can pick up things very well. (P2, CP)*
	Stress management	*Stress management, 2020 batch, so far, I did not hear any complaints. I did not hear any complaints from other units. 2019 batch got, but 2020 no. But I would have to say that stress management is very personal, it is very individualized. I feel that it has nothing to do with the presence of COVID [pandemic] or not, whether they are doing online study, because from the experiences that I had with PRPs with problems, is I feel there is nothing to do with their university or their college or whatever. I feel it is very individualized [......] (P6, GH)*
Organizational acumen	–	*Workplace policy in terms of if you're comparing batches to batches, it is about the same because every workplace will have a different policy. (P1, CP)* *Company policy wise, not much difference. Both batch they understand quite well, the organization workflow, the organization chart, how we deal [with things]. (P4, CP)* *Over time they will definitely get better, in terms of our operation procedures, whatever they need to do, most of the time they will improve. But the difference no, in terms of the cohorts. (P9, CP)*
Impact on career choice	–	*For the pandemic batch, I am concerned because they didn't have attachment in hospital or community. During interview, they do not know whether they prefer to be in the community pharmacy, hospital or industrial….. I found that graduates don't know their direction well and they didn't have any experience in terms of what to choose for their pathway or future. (P2, CP)*

**Table:** 

Participant ID	Demographic (Age, Gender, Graduating Year)	Theme	Sub-theme (direction)	Quote
S1	25, male, 2020	Experiences (on-campus)	Missing out – relationships (negative)	I feel there are more cases to learn and colleagues are closer to each other
S2	24, female, 2020	Experiences	Equity (positive)	I feel Covid 19 made everything fair and easy as the focus on the pandemic. It eases “face problem” […] since everyone is wearing the mask
S3	24, female, 2020	Experiences (placements)	Missing out – learning settings (negative)	If there was no covid, I would have been able to finish my hospital attachment during my final semester. Since I'm working as a PRP [provisionally registered pharmacist] in a community pharmacy now, I would feel more comfortable talking and giving counselling to the customer for a longer time if there was no covid because right now we need to stay cautious and reduce contact time with customers.
S4	23, female, 2020	Experiences (on-campus)	Face-to-face interaction (negative)	Better real life skills (PBL (problem based learning) virtually is not the same as PBL in a class with your groupmates)
S5	24, female, 2020	Experiences (placements)	Work-readiness (negative)	Although the difference is not significant, I believe that the clinical attachment opportunities that we missed out on during the COVID-19 pandemic would have prepared us better for the fast-paced work setting.
S6	24, female, 2020	Experiences (On-campus)	Lab technical skills (positive and negative)	Experiences gained from clinical placement is very subjective to sites/preceptors involved and hence, the pandemic might not affect much on work readiness Campus learning activities, even when switched online, were still as fruitful and enjoyable. The only downfall was the lack of actual extemporaneous preparation activities which were limited by the pandemic
S7	23, male, 2020	Experiences (On-campus)	Work readiness (negative)	COVID-19 impedes the human touch among pharmacists and patients, rendering counselling to be not as effective as it used to be. Face shields softens pharmacists' voices and patients are not allowed to demonstrate the usage of devices such as inhalers and nasal sprays in certain conditions. This overall may lead to ineffective counselling. Besides, video counselling is being promoted widely but no proper guidelines about effective counselling have been provided. This overall may impact my work readiness. If there is no COVID-19, I could have done better and aim for higher goals.
S8	24, female, 2020	Experiences	Employment opportunities (negative)	If there is no MCO (movement control orders), I am able to work with paid OT (overtime). I would have more exposure to my current field of career.
S9	24, female, 2020	Experiences (on-campus & placements)	Choice of employment setting (negative) Virtual learning – retention of learning	I would know which field I'm more interested to if clinical attachment proceeded as per normal (I'm a Monash fresh grad and I'm lucky that I manage to get hospital attachment and “klinik kesihatan” (government health clinic) attachment before the first MCO was implemented in 2020, hence it helps me in making my career decision) I feel the learning activities will be better if everything proceeded as normal. I spent my whole Year 4 learning online and I found that I tend to forget the clinical knowledge that I learnt in year 4. As for the clinical knowledge that I learnt in year 2 and 3, somehow I can remember more.
S10	24, female, 2020	Experiences	Personal development (negative)	If there is no covid 19 pandemic, it will be follow my plan and i would joining some activities which can improve my personal skills.
S11	26, female, 2020	Work conditions	Customer expectations (negative)	There will be lesser stress in terms of fulfilling customer's needs on fast-selling COVID-19 items (face shield, sanitizer, face mask, COVID-19 test kits, etc.).
S12	24, female, 2020	Experiences (placements)	Exposure to various work settings and influence of career choice (negative)	If there was no covid pandemic, I would have gotten more exposure to clinical attachment in hospital and government clinic during my studies. It may change my decision on working in a retail pharmacy. Also, the services provided in retail pharmacy may differ from the situation now.
S13	25, male, 2020	Experiences (placements)	Equivalent virtual activities (negative) Technology adoptions (positive)	We are supposed to attached to government hospital/clinics during my final year, but we failed to due to the pandemic. All the clinical attachments was forced to run online mode and that really made me struggle during my PRP training. During my training, we as prp are restricted from doing in ward medication history taking and counselling. The bright side of it, we are all running in digital format. (electronic data/video counselling)
S14	24, female, 2020	Adaptable		Ready to adapt the environment
S15	24, female, 2020	Experiences (on-campus)	Campus experience contributing to work readiness (negative)	Being able to meet people physically on campus would have allowed more informal conversations about areas that I did not even know about that could better help work readiness.
S16	22, male, 2021	Experiences (placement)	Clinical experiences (negative)	I would have greater opportunity in clinical attachments, thus predicted to have better work readiness especially in clinical field.
S17	23, male, 2021	Experiences (placement & on-campus)	Lab skills (negative) Clinical application (negative)	I believe I could be even more competent, in term of lab skills and also application of clinical knowledge if I were given the opportunity to carry out lab project and also have hospital attachment during my course.
S18	23, female, 2021	Experiences (on-campus)	Communication (negative)	I personally think that there might be a slight different as if there was no COVID-19, we would have 100% physical classes which require ongoing f2f communication that somehow strengthen the oral communication skill. COVID-19 related self-isolation may weaken one's oral communication skills.
S19	22, female, 2021	Experience (on-campus)	Understanding (negative)	More hands on practice. Hence, higher understanding.
S20	22, female, 2021	Experiences (placements and on-campus)	Professional development (positive) Influence on choice of work setting	I look at the pandemic positively. Because of the lack of clinical attachments, I have more time to take up internship and WFH. Online study also gives me more time to join more events, clubs without having to travel and attend those events physically which saved up a lot of time. Furthermore, work interviews, meetings are all conducted virtually now which is much more convenient and less nerve wrecking compared to F2F. I really love the situation now, I hope virtual meeting/class can be continued. However, bc of the lack of clinical attachments, I'm swayed away from working in hospital.
S21	22,female, 2021	Experiences (placements)	Confidence Clinical application	I think I would be more prepared and confident as I step out to work if our placements are arranged as per usual without the pandemic. Honestly placements really help to gain better insights and allow us to immerse in real life settings. Absence of placement makes it quite hard to apply theory to practice as we don't see real life cases

**Table C1:** Table of demographic details of participants who took part in survey.

	**Number**	**Frequency (%)**
Gender		
Male	5	16.7
Female	25	83.3
Ethnicity		
Malay	3	10.0
Chinese	26	86.7
Indian	0	0
Others	1	3.3
Pre-University background		
A-Level	12	40.0
MUFY	5	16.7
STPM	2	6.7
CPU	0	0
Others (eg. UEC, Foundation course)	11	36.7
Graduation Year		
2020	21	70.0
2021	9	30.0
Started provisionally-registered pharmacist training		
Yes	20	66.7
No	8	26.7
No, but doing something else	2	6.7
Workplace country		
Malaysia	27	90.0
Overseas	0	0
Not relevant	3	10.0

SD = Standard Deviation.

MUFY = Monash University Foundation Year.

CPU = Canadian Pre-University.

STPM = Sijil Tinggi Persekolahan Malaysia (Malaysian High School Certificate).

UEC = Unified Examination Certificate.

**Table:** 

**Questions**	**% Disagree**	**% Agree**
**Block 1**		
1. I am sometimes embarrassed to ask questions when I am unsure about something.	40	60
2. I maintain an appropriate balance between work and non-work interests.	20	80
3. I am able to switch off when I am not at work.	53.3	46.7
4. I know my strengths and weaknesses.	30	70
5. I am passionate about my field of study or work	16.7	83.3
6. It is important to respect authority figures	13.3	86.7
7. When a crisis situation that needs my attention arises I can easily change my focus	16.7	83.3
8. I set high standards for myself and others	13.3	86.7
9. I thrive on completing tasks and achieving results	6.7	93.3
10. I do not take patients' or clients' aggressive behaviour personally	33.3	66.7
11. I feel confident to address interpersonal conflicts at work	53.3	46.7
12. I feel confident to ask for support in dealing with interpersonal conflicts at work	36.7	63.3
13. It is important to respect your colleagues	0	100
14. I sometimes have difficulty starting tasks	50	50
15. Being among the best in my field is very important to me	30	70
16. At work it is important to always take responsibility for your decisions and actions.	0	100
17. Ability to juggle many things at once is one of my strength	36.7	63.3
18. I am able to deal with things when there are competing demands on me	26.7	73.3
19. I do not like learning new things	93.3	6.7

**Block 2**		
20. I do not like the idea of change	66.7	33.3
21. I get stressed when there are too many things going on	3.3	96.7
22. I remain calm under pressure	70	30
23. I know how to cope with multiple demands	33.3	66.7
24. I am confident about my learnt knowledge and could readily answer technical questions about my field	43.3	56.7
25. I have a solid theoretical understanding of my field of work	36.7	63.3
26. One of my strengths is that I have is an eye for detail	33.3	66.7
27. I feel confident that I will be able to apply my learnt knowledge to the workplace	20	80
28. Now that I have completed my studies I consider myself technically competent to apply myself to the field	36.7	63.3
29. As a graduate, listening and learning are more important than showing your knowledge	10	90
30. What is happening in the world can have a great impact on business decisions	3.3	96.7
31. As an employee it's important to have a sound understanding of organizational processes and protocols	0	100
32. One of my strengths is developing relationships with people.	16.7	83.3
33. I can express myself easily	43.3	56.7
34. I am good at making impromptu speeches	66.7	33.3
35. I am good at reading other people's body language	13.3	86.7
36. I communicate effectively with different patients or clients	16.7	83.3
37. Others would say I have an open and friendly approach	16.7	83.3
38. Working in groups is one of my strengths	26.7	73.3

**Block 3**		
39. You can learn a lot from long serving employees, even if they do not have a university degree	0	100
40. Adapting to different social situations is one of my strengths	20	80
41. I adapt easily to new situations	30	70
42. Analyzing and solving complex problems is one of my strengths	33.3	66.7
43. I consider myself to have a mature view of life	23.3	76.7
44. An organization's values and beliefs forms part of its culture	0	100
45. There is a lot to learn from employees who have worked at an organization for years	3.3	96.7
46. It is important to learn as much as you can about the organization	3.3	96.7
47. It is important for employees to keep up with current work affairs	10	90
48. I look forward to the opportunity to learn and grow at work	6.7	93.3
49. I am eager to throw myself into my work	30	70
50. I see all feedback as an opportunity for learning	3.3	96.7
51. Graduates need to be willing to start at the bottom and work their way up	10	90
52. I become overwhelmed by challenging circumstances	33.3	66.7
53. I find it easy to approach senior people at work	43.3	56.7

## Appendix B

**Pharmacy students response to** ‘Please comment on how your work readiness would have differed, if there was NO Covid-19 pandemic and learning activities/clinical attachments proceeded as per normal” via free-text box

## Appendix C

**Results of survey responses from Work-Readiness Scale**

## Appendix D – Interview Guide

1. From your experience of supervising pharmacy graduates in their first year of work, please share your thoughts on work readiness. (Ask the interviewee to define their understanding of work readiness)
2. In your XX years of work, what are the important points that you feel need to be addressed in terms of the work readiness of new graduates.
3. Specifically, could you contrast your experiences of working with the graduating batch affected by the pandemic compared with those before?
4. When thinking about work readiness for the different batches, please consider these 4 factors – work competence, social intelligence, personal characteristics and organizational acumen.
